# Multidisciplinary and home-based management in neonatal unilateral foot drop: a case report

**DOI:** 10.3389/fresc.2025.1575708

**Published:** 2025-05-20

**Authors:** Ilaria Sanzarello, Lorenza Siracusano, Angelo Alito, Carmela De Domenico, Matteo Nanni, Danilo Leonetti

**Affiliations:** ^1^ Department of Biomedical, Dental and Morphological and Functional Images, Section of Orthopaedic and Traumatology, University of Messina, Messina, Italy; ^2^ IRCCS Centro Neurolesi Bonino Pulejo, Messina, Italy

**Keywords:** drop foot, external popliteal sciatic nerve, neuropathy, multidisciplinary management, neonate

## Abstract

**Background:**

Foot drop in newborns is a rare condition with limited cases reported in the literature. It can result from various aetiologies including neurological, muscular, anatomical, or mechanical factors. Diagnosis can be challenging as identifying the underlying cause is essential for determining the appropriate course of management.

**Case presentation:**

We present the case of a newborn with unilateral foot drop highlighting the diagnostic approach and clinical progression. Clinical evaluation and instrumental examinations, including electromyography and nerve conduction studies, showed isolated external popliteal of the sciatic nerve dysfunction. There were no associated spinal cord or musculoskeletal abnormalities. A prolonged and complicated delivery, with sustained intrauterine limb malposition and nerve compression, was identified as the likely cause, leading to transient ischemia and peripheral nerve impairment. Despite the initial weakness and inability to dorsiflex the foot, no surgical or pharmacological intervention was required. Supportive care and close clinical monitoring were adopted besides the active involvement of parents in foot positioning and mobilization. Over the following months, gradual neurological recovery was observed, culminating in the complete resolution of symptoms by the fifth month of life.

**Conclusion:**

This case underscores the importance of recognizing transient peroneal nerve palsy as a potential cause of neonatal foot drop. It also highlights the role of conservative management and expectant observation in cases where spontaneous recovery is likely, avoiding unnecessary interventions.

## 1 Introduction

Foot drop is described as the inability to perform dorsal flexion of the ankle and eversion of the foot, associated with static supination, which may be due to either neurological, muscular, or anatomical disorders (1). The most common cause of foot drop is an alteration of the external popliteal sciatic nerve (EPSN) function, which is responsible for the activation of the ankle dorsiflexor and foot evertor muscles [tibialis anterior, extensor hallucis longus (EHL), extensor digitorum longus, and peroneus longus, brevis, and tertius] (2, 3). EPSN neuropathy may result from many causes, such as trauma, vascular or neurological injuries/diseases, tumors, and congenital malformation.

Foot drop in the newborn is rare. The prevalence of EPSN neuropathy among lower limb neuropathies in pediatric patients is particularly notable, accounting for 59% of cases, followed by the involvement of deep (12%) and superficial (5%) peroneal nerves. Regarding neonatal foot drop, there are only a few reports in the literature, and the pathological mechanisms are not fully understood. However, it is usually associated with birth trauma or nerve compression due to prenatal causes (4–6). Neonatal EPSN neuropathy usually is characterized by a clear postnatal onset. Possible causes included uterine contractions, amniotic bands, footboard compression, and, more commonly in the past, compartment syndromes due to intravenous fluid infiltration or umbilical arterial injection of vasoconstricting agents or hypertonic solution (3, 7, 8). Primiparous mother, breech presentation, or prolonged delivery leading to cesarean section are prenatal risk factors for peroneal neuropathy (9, 10).

The assessment of foot drop should begin with a physical examination to identify static supination due to the inability to perform dorsal flexion of the ankle and eversion of the foot. Several clinical scores can be used, such as the Hammersmith Infant Neurological Examination (HINE) or the Amiel–Tison Neurological Assessment (ATNA) (11–13).

Instrumental investigations are required to rule out structural causes involving the bones, nerves, and tendons surrounding the ankle joint and to individualize the underlying cause of the condition. Brain and spinal imaging may be considered if indicated by clinical findings. However, the most significant investigations to consider are electromyography (EMG) and nerve conduction studies (NCS), although these are difficult to perform in newborns and may require sedation (14–16).

If a patient's results are negative on routine testing, further diagnostic procedures may be required. These may include genetic testing, which can be utilized to evaluate conditions such as multiple sclerosis, spinal muscular atrophy (SMA), and congenital neuropathies or dystrophies (17, 18).

Treatment of foot drop varies depending on the underlying cause; indeed, a thorough assessment of the patient is necessary before deciding on a treatment strategy. Non-surgical treatments include close monitoring, physiotherapy, and the use of orthotics to prevent foot drop. Otherwise, surgical procedures such as neurolysis and nerve or tendon transfer may be required (19). Supportive care, such as gentle exercise and physiotherapy, can help maintain joint mobility and prevent muscle stiffness while waiting for nerve function to return (20). Parents may be instructed to encourage normal leg movements through positioning techniques and supervised exercises. If there is no improvement after several months, or if imaging shows structural nerve damage, specific treatments such as nerve stimulation therapy or, in extreme cases, surgical nerve decompression or tendon transfer may be considered.

This work aims to describe the role of multidisciplinary and home-based management in addressing newborn foot drop, through the presentation of a rare case of isolated foot drop in a healthy newborn who fully recovered within a 5-month period. The rationale behind this article is its infrequency, which can lead inexperienced doctors to misdiagnose.

This case report followed the CARE guidelines for case reports (21).

## 2 Case presentation

The patient is a female infant born in October 2022, at 38.0 weeks' gestation to a 35-year-old primiparous mother who underwent in vitro fertilization (IVF) treatment. No complications during pregnancy nor relevant maternal or paternal pathological history were recorded. The child had a cephalic presentation, and labor was induced and 36 h long; hence, she was delivered by caesarean section. She weighed 3.620 kg and was 52 cm long. Apgar scores were 7 and 9 at 1 and 5 min, respectively, due to meconium-stained fluid.

**Figure 1:**
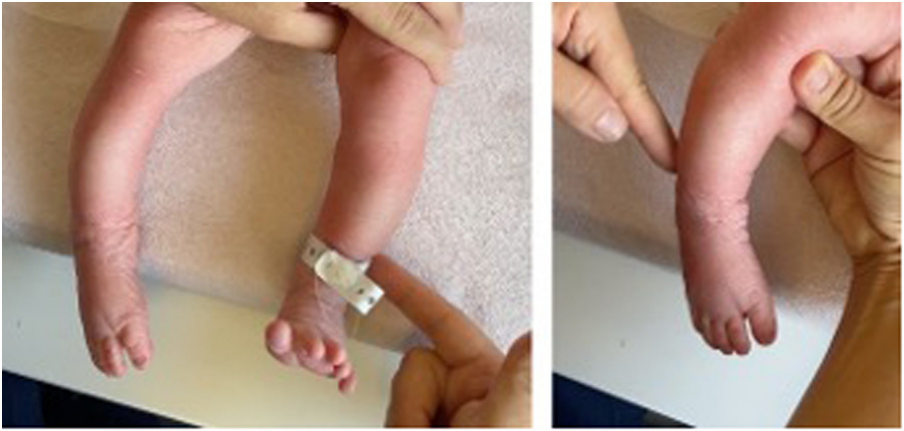
**(A)** Physiological response to tactile stimulation on the left side with consequent ankle dorsiflexion. **(B)** Foot drop on the right side with no response to either tactile or pain stimulation.

At birth, the general physical examination was normal, except for the legs, which had different skin folds, with those on the left more prominent than those on the right. Skin color also appeared different, with marked cyanotic coloring in the right lower limb*.* Neurological examination was performed with HINE, an assessment tool widely used in both clinical and medical research to discriminate at-risk newborn infants. Orientation and behavior of the newborn term infant were normal. The spontaneous posture exhibited by the patient was characterized by flexion and external rotation of the arms. The left leg exhibited a similar alignment, demonstrating flexion and external rotation. Conversely, the right leg exhibited minimal flexion and abduction. Tone patterns were physiological, except for the right lower limb, which exhibited hypotonia, particularly distally. Upon abrupt passive extension of the legs, the right leg did not show a physiological reaction, with an extreme reduction in movement of the leg and foot. Spontaneous movements of the right leg appeared reduced compared with the left side while spontaneous movements of the right foot were absent. Flexion of the right thigh on the trunk could only be elicited by painful tactile stimulation and was abrupt and jerky. Grasping reflexes were found to be physiological except for the right foot. A paresis of the extensor, dorsiflexor, and evertor muscles was detected indicating a stupor of both the tibial nerve and EPSN resulting in a paretic equinovarus, supinated foot without tendinous retractions (Figure 1, Supplementary Video 1). After tactile stimulation, there was weak foot eversion and toes dorsiflexion, except for the hallux. Peripheral limb circulation appeared to be preserved. Twelve hours postnatally, edema observed at birth resolved spontaneously without the need for medical intervention. Overall, the collected clinical and neurological signs suggested an intrauterine malposition, probably worsened by fetal distress.

In the following days, many investigations were conducted to rule out possible metabolic, muscular, genetic, and neurologic disorders. The echocardiogram and abdominal ultrasound were normal. The spine and the cranium ultrasounds showed a fatty and thickened appearance of the filum terminale; therefore, an MRI (magnetic resonance imaging) was performed which showed no pathological findings. To assess peripheral nerve function, a first electromyography (EMG) was performed 5 days after birth which supported the diagnosis of isolated EPSN mononeuropathy, while muscle action potential (MAP) and nerve conduction velocity (NCV) were attempted, but they could not be recorded with surface electrodes only. In addition, a genetic analysis was performed that revealed mosaicism of a deletion in chromosome 5 (5q13.1q13.2) involving 47 genes, including the survival motor neuron 1 (SMN1) gene, which is associated with spinal muscular atrophy (SMA). However, this variant is not currently associated with any pathologic manifestation, as confirmed by genetic counseling.

During hospitalization, the child was treated conservatively by the ward staff with manipulation and tactile stimulation of the right lower extremity involving knee, ankle, and foot mobilization. In addition, parents were taught by a physiotherapist and the pediatric orthopedic surgeon to passively move the foot to assess possible changes in its flexibility and to avoid the development of muscle or joint capsule retraction. They were also instructed to promote active movement of the foot by touching, tickling, and pinching the dorsal and plantar skin on the lateral side of the leg and foot. After discharge, physiotherapy performed by the caregivers was the only treatment administered, and no medication, orthosis, or electrotherapy was prescribed.

After 1 month, the patient underwent a clinical and neurological examination assessed with HINE and an EMG. There were improvements in tone patterns, reflexes, movements, and behavior, but movements were not yet in line with age expectations. She was able to spontaneously dorsiflex and evert the foot and was responsive to stimulation. On the other hand, the deficit of the EHL muscle persisted, showing an activation only after pain stimulation. The EMG showed no evidence of denervation of the peroneal nerve and the conduction velocity improved compared with the previous exam. Parents were instructed to continue to perform manipulations and tactile stimulations on their child and were further advised to provide weekly feedback to the attending physicians, in preparation for the next ambulatory visit.

**Figure 2:**
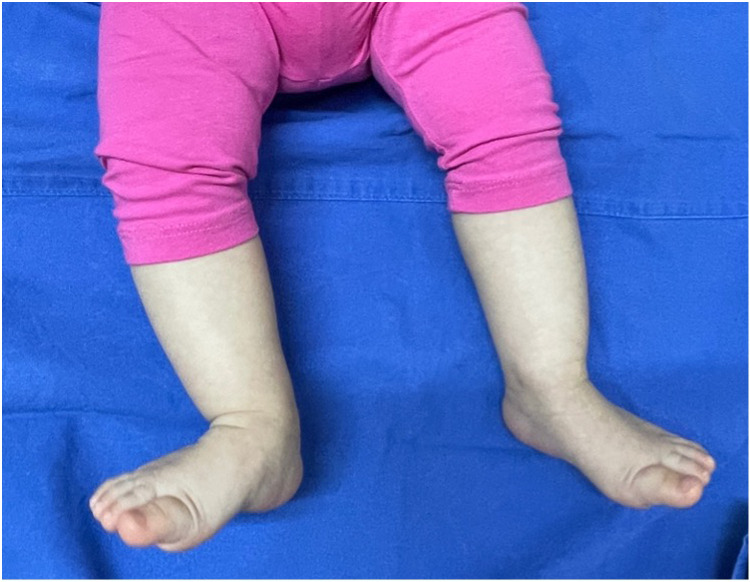
Recovered active ankle dorsiflexion and foot eversion after 5 months.

**Figure 3:**
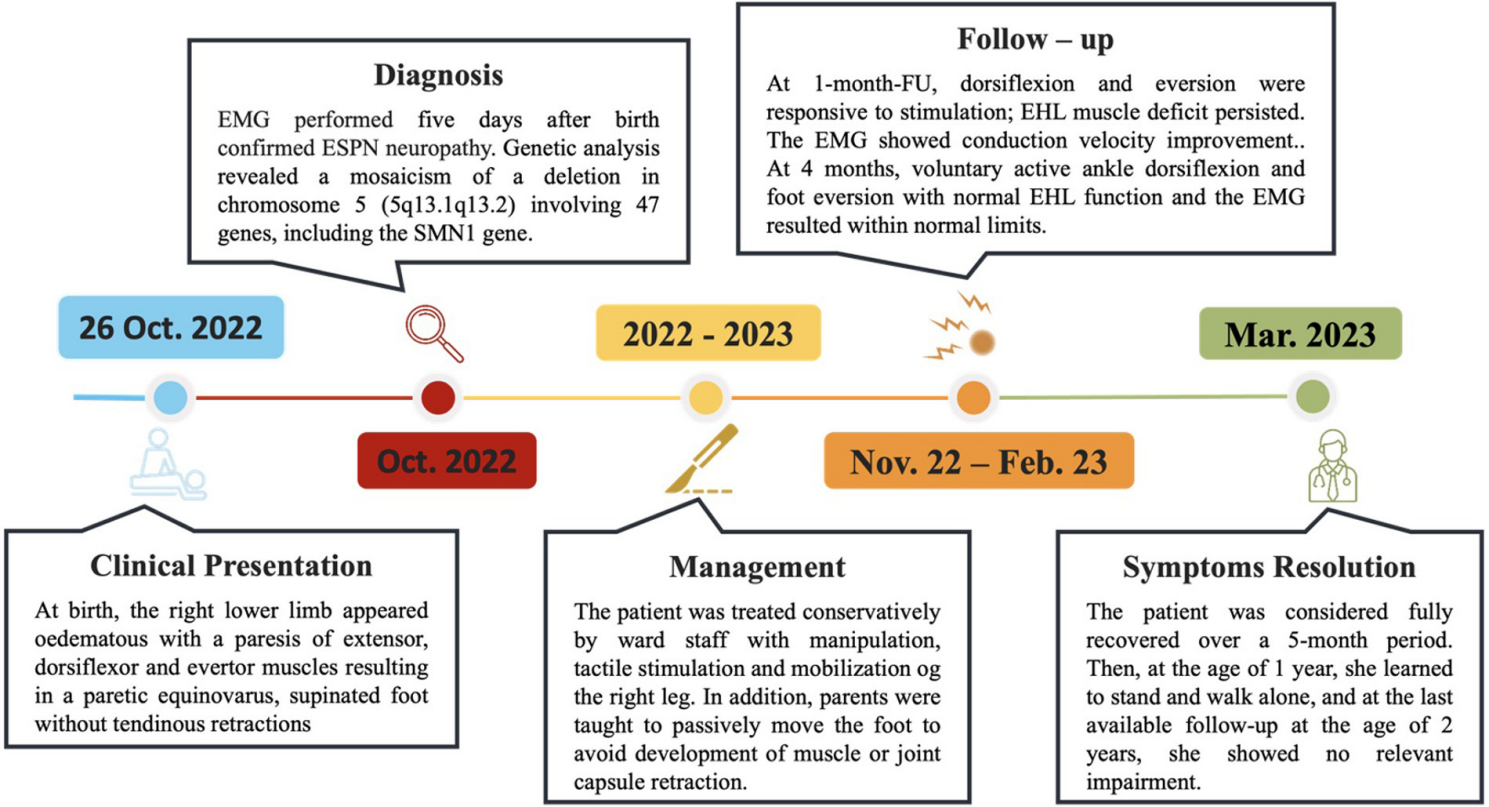
Diagnosis and treatment timeline of the patient. The timeline shows the major clinical events during the patient's management. *Template by PresentationGO, www.presentationgo.com (accessed on 03 February 2025).

At the 4 months of follow-up, the physician observed voluntary active ankle dorsiflexion and foot eversion with normal EHL function, and the electrophysiological study resulted within normal limits (MAP = 6 mV; NCV = 32 ms) according to her age (14, 22). The patient was considered fully recovered over a 5-month period (Figure 2). Then, at the age of 1 year, she learned to stand and walk alone, and at the last available follow-up at the age of 2 years, she showed no relevant impairment (Figure 3).

## 3 Discussion

Mononeuropathies in newborns are quite uncommon. Most frequently they involve the brachial plexus, radial nerve, or facial nerve, but lower limb isolated neuropathies have been described as well. Among them, the most common is EPSN deficit resulting in isolated congenital foot drop (23).

Primiparous mother, breech presentation, and long or complicated labor followed by cesarean section have been reported as possible risk factors. EPSN neuropathy can also result from birth traumas, congenital disorders, or even intrauterine causes (2, 24, 25). Regarding intrauterine prenatal onset, this is usually related to compression mechanism, for instance, in the case of amniotic bands, uterine ring contractions, fetal mispositioning, or procedures no longer performed such as umbilical vessel injections, as reported by some authors (4, 26). These conditions may lead to impairment of the lower limb circulation, hence ischemia, that ultimately produces nerve suffering and neuropraxia or even more severe neurologic damage. In view of the multiple potentially associated etiologies, the employment of specific and targeted tests would be advantageous to narrow down the hypotheses and to detect or exclude possible serious pathologies. At present, however, electrophysiological and nerve conduction studies are still the main, and almost always the only, tests performed for diagnosis and follow-up (4, 6).

Treatment depends on the underlying cause. Therefore, multidisciplinary management may be required depending on etiology. However, in the case of foot drop due to isolated EPSN neuropathy, according to literature data, the prognosis is generally favorable and early physiotherapy and/or mobilization are sufficient to reach a positive outcome (27, 28). Obviously, parents/caregivers are an essential part of the multidisciplinary team, and they need to be involved and educated about their newborns’ status, as we have shown in this case report in which parents were the ones administering mobilization therapy to the patient, providing weekly feedback to the attending physicians. As primary caregivers, parents play a vital role in ensuring compliance with treatment plans, including physical exercises, holding, and handling maneuvres and follow-up appointments. They are the primary source of information about the baby's environment, behavior, and daily routines, which is essential for the development of an individualized care plan (28). Their active participation in home-based interventions, along with ongoing education and support from the medical team, enhances the effectiveness of treatment and promotes optimal psychological and motor development (26).

This report describes a rare case of isolated foot drop in a primiparous mother who had a long birth labor followed by a cesarean section. Immediately after birth, the infant presented with an oedematous lower right limb, suggesting prolonged intrauterine malposition and limb compression. Although vascular disease is considered a primary cause of limb edema, fortunately, no relevant acute peripheral vascular compromise of the limb was detected at birth. As the initial EMG was positive for EPSN neuropraxia, transient limb ischemia with neurological distress was suspected. This was our primary suspect; subsequently, we performed the necessary testing to rule out other conditions, such as vascular or neuromuscular diseases.

After discharge, in addition to close monitoring, only conservative treatment was recommended, and it was carried out by the parents, with no defined treatment protocol in terms of time and frequency. Parents were instructed to perform daily passive mobilization of the ankle and foot, appropriate positioning during rest and care routines, and tactile stimulation to encourage active movement were performed. The parents reported that they consistently applied these techniques at home throughout the follow-up period, and the progressive clinical improvement observed at each follow-up proves active parental involvement.

Regular follow-up is necessary to assess the evolution of the condition helping to evaluate symptoms and adjust treatment accordingly. Moreover, regular assessment helps to implement timely corrective measures such as bracing or stretching exercises. Follow-up visits support and educate parents about home exercises, positioning techniques, and the expected progression of healing, helping to reduce anxiety and ensure compliance with treatment plans.

At each follow-up, the patient showed progressive improvement both clinically and in the electrophysiological pattern. Finally, at 5 months, she was considered fully recovered.

Finally, our experience is limited, but current literature confirms that isolated neonatal foot drop is associated with a good prognosis, often resulting from intrauterine compression. On the other hand, in the case of a newborn who presents with EPSN neuropathy, potentially serious conditions must be ruled out. This paper has some limitations. As a single case, it lacks generalisability and does not establish a causal relationship between the factors identified and the disease. Despite these limitations, this report highlights the importance of early diagnosis and follow-up, contributes to the existing evidence, and guides further research into neonatal foot drop.

The scarcity of literature with a lack of prospective studies and the small sample size give rise to a considerable risk of misdiagnosis or diagnostic delay. Further studies are required to expand our knowledge of this rare condition, with a view to improving diagnostic guidelines and training inexperienced physicians.

## 4 Conclusion

Foot drop in newborns is a rare condition with variable aetiologies and associated therapies. There are a few cases in the literature describing isolated congenital EPSN and subsequent foot drop with complete recovery without prolonged or even surgical treatment. However, they all agree on the possible pathological mechanisms and risk factors. Congenital isolated foot drop must be carefully differentiated from other systemic or local disorders. A multidisciplinary approach plays a key role in the management of neonatal foot drop, involving neonatologists, neurologists, physiotherapists, and orthopedic specialists to ensure comprehensive care. Early diagnosis, regular follow-up, and coordinated interventions are essential to optimize recovery and prevent long-term complications.

## Data Availability

The raw data supporting the conclusions of this article will be made available by the authors, without undue reservation.

## Supplementary material

Supplementary Video 1 Video showing both tibial nerve and EPSN suffering resulting in a paretic equinovarus, supinated foot without tendinous retractions a few days after birth.
